# Surgery for Tetralogy of Fallot in Adults: Early Outcomes

**DOI:** 10.5935/1678-9741.20160063

**Published:** 2016

**Authors:** Imran Khan, Zafar Tufail, Saeed Afridi, Madiha Iqbal, Tipu Khan, Abdul Waheed

**Affiliations:** 1 Punjab Institute of Cardiology, Lahore, Pakistan.

**Keywords:** Tetralogy of Fallot, Adult, Hospital Mortality

## Abstract

**Objective:**

To study the in-hospital outcome of adult patients who had undergone surgical repair for Tetralogy of Fallot.

**Methods:**

A retrospective descriptive study was conducted at the Punjab Institute of Cardiology searching the hospital records. All those adult patients who had undergone repair for Tetralogy of Fallot from January 2012 to December 2014 were included in the study. All the patients were operated by the same surgical team. Patients who underwent primary repair as well as those with previous palliative procedures were included in the study. Thirty days outcome was studied by recording variables from the database. Data was analysed using Statistical Package for Social Sciences version 16.

**Results:**

A total of 80 patients was included in the study, in which there were 48 (60%) male patients and 32 (40%) female patients. Mean age was 21±0.21 years. Those with previous palliation were 15 (18.75%). The associated problems observed were: atrial septal defect 27 (33.75%), right aortic arch 30 (37.5%), patent ductus arteriosus 6 (7.5%) and double outlet right ventricle 3 (3.75%). In-hospital mortality recorded was 7 (8%). Postoperative complications encountered were low cardiac output syndrome 9 (11.25%), pleural effusion requiring tapping 3 (3.75%), reoperation for bleeding 3 (3.8%), pulmonary regurgitation (moderate to severe) 20 (25%) which occurred in the transannular patch group only and atrial arrhythmia 4 (5%).

**Conclusion:**

A large number of adult patients are still operated for tetralogy of Fallot in Pakistan. With increasing experience in the technique the mortality and morbidity is comparable to international literature.

**Table:** 

Abbreviations, acronyms & symbols	
AVSD	=Atrioventricular septal defect
LAD	=Left anterior descending
MAPCAS	=Major aortopulmonary collateral arteries
PA	=Pulmonary artery
PR	=Pulmonary regurgitation
RCA	=Right coronary artery
RV	=Right ventricular
RVOT	=Right ventricular outflow tract
TAP	=Transannular patch
ToF	=Tetralogy of Fallot
VSD	=Ventricular septal defect

## INTRODUCTION

Paediatric cardiac surgery started in Pakistan in the 80's. Since then our competencies in this particular field of cardiac surgery have improved a great deal. Because of the comparatively recent introduction of congenital cardiac surgery in Pakistan, the surgery of congenital cardiac problems in adults has been an area of interest in many centers in the country.

Tetralogy of Fallot (ToF) is the most frequent cyanotic congenital heart disease, constituting 7 to 10% of all the congenital heart diseases^[1]^. It carries a very high mortality if left untreated. The 10-year survival in patients untreated is 24% only^[2]^. The basic anatomical defects stems from the anterior and superior deviation of the infundibular septum which results in the four main features of the disease i.e. subvalvar pulmonary stenosis with hypoplasia of pulmonary artery (PA) valve and pulmonary arteries, non-restrictive malalignment sub-arterial ventricular septal defect (VSD), overriding aorta and right ventricular (RV) hypertrophy. Cyanosis is the main physical feature of the disease whose severity is determined by the degree of right ventricular outflow tract (RVOT) obstruction stenosis, which determines the right to left shunt. Associated anomalies are secundum atrial septal defect, right aortic arch (25%), major aortopulmonary collateral arteries (MAPCAS), complete atrioventricular septal defect (AVSD), anomaly of coronary arteries with *e.g*. anomalous left anterior descending (LAD) from right coronary artery (RCA) crossing the RVOT (5%)^[3]^. Cardiac magnetic resonance is the gold standard assessment tool based on its superior imaging of the RVOT, pulmonary arteries, aorta, and aortopulmonary collateral arteries, and on its ability to quantify biventricular size and function, pulmonary regurgitation (PR), and myocardial viability^[4]^. Primary repair is the strategy of choice where possible in the first year of life. Patients with unfavourable anatomy, comorbidities and severe cyanosis undergo a palliative shunt procedure before the definitive corrective surgery later on in life.

The purpose of this report was to know the outcome of corrective surgery for ToF in our setup in terms of short-term mortality and morbidity. To the best of our knowledge, this is the largest report so far about ToF repair in adult patients in Pakistan.

## METHODS

A retrospective descriptive study was designed to study the in-hospital outcome of all the adult patients who had undergone surgical correction for ToF from January 2012 to December 2014. The hospital database of the Punjab Institute of Cardiology was searched for this purpose and patient variables were recorded. Being a retrospective study, individual patient consent was waived by the hospital ethical review committee.

The basic surgical steps were identical in all the patients *i.e.* use of cardiopulmonary bypass and avoiding ventriculotomy in most patients. Patients who had undergone palliative procedure previously were evaluated for hemodynamic parameters and size of PA. Those without any irreversible pulmonary arterial hypertension and unfavourable pulmonary anatomy were subjected to final corrective procedure. The goal of repair was a complete relief of RVOT and closure of VSD. Patients with only subpulmonic narrowing underwent infundibular resection of the myocardium. Patients with narrowing of the pulmonary valve annulus also underwent pulmonary valvotomy or placement of a transannular patch (TAP). In these cases, a relief of pulmonary obstruction was achieved at the cost of varying degrees of pulmonary insufficiency.

Perioperative variables were recorded and data analysed using Statistical Package for Social Sciences version 16. Quantitative variable were presented as mean ± standard deviation and the qualitative variables were presented as frequency and percentages. For the comparison of the quantitative data, independent sample t-test was applied while for qualitative data chi-square was used. A *P*-value ≤ 0.05 was considered significant.

## RESULTS

A total of 80 patients were included in the study. Most of the patients included were male, 48 (60%), while female patients were 32 (40%). Preoperative characteristics are shown in Table 1. Mean age of the patients was 21±0.21 years with a range of 12 to 43 years. Patients were divided into 3 based on whether they received a TAP, only RVOT patch or no patch at all. Among the 80 patients, 15 patients had already undergone a palliative surgical procedure so they underwent a complete corrective surgery or two-stage procedure. Different morphological forms identified in our patients were: main PA stenosis 24 (30.0%), right PA stenosis 14 (17.5%), left PA stenosis 12 (15%), MAPCAS 5 (6.25%), right aortic arch 30 (37.5%), aberrant subclavian artery 3 (3.75%), atrial septal defect 27 (33.75%), left superior vena cava 5 (6.25%), patent ductus arteriosus 6 (7.5%), and double outlet right ventricle 3 (3.75%). A right aortic arch, atrial septal defect and main PA stenosis were the most common associated malformations observed. No pericardial RVOT or TAP was used in 23 (28.75%) patients. TAP for narrow pulmonary annulus was used in 29 (36.25%) and RVOT patch was used in 28 (35.0%) patients. Most of the patients underwent a patch enlargement of infundibular-pulmonary outflow tract. Complications noted in the postoperative period were low cardiac output syndrome 9 (11.25%), heart block 4 (5.0%), pleural effusion requiring tapping in 3 (3.75%), atrial arrhythmia 4 (5%), reoperation for bleeding 3 (3.8%) and PR (moderate to severe) 20 (25%). Mortality observed during the 30 days in the postoperative period was 7 (8%) (Table 2).

**Table 1:** Patient characteristics.

Characteristic		Number of patients	No patch	Transannular patch	RVOT patch	*P*-value
		80 (100%)	23 (28.75%)	29 (36.25%)	28 (35.0%)	
Mean age (range; 12-43) year		21±0.21	14±0.32	17±0.41	19±0.44	0.001§
Gender	Male	48 (60%)	15 (65.2%)	16 (55.2%)	17 (60.71%)	0.182#
Previous palliation		15 (18.75%)	6 (40.0%)	5 (33.3%)	4 (57.14%)	0.035
Morphology						
Main pulmonary artery stenosis		24 (30.0%)	7 (30.43%)	9 (31.03%)	9 (28.57%)	0.059
Right pulmonary artery stenosis		14 (17.5%)	3 (13.0%)	5 (17.8%)	6 (20.6%)	0.033
Left pulmonary artery stenosis		12 (15%)	2 (8.6%)	4 (13.7%)	6 (20.6%)	0.002
Major aortopulmonary collateral arteries		5 (6.25%)	1 (4.3%)	2 (6.8%)	3 (10.6%)	0.028
Right aortic arch		30 (37.5%)	7 (30.43%)	10 (34.48%)	13 (46.42%)	0.001
Aberrant subclavian artery		3 (3.75%)	1 (4.34%)	1 (3.57%)	1 (3.45%)	1.457
Atrial septal defect		27 (33.75%)	6 (30.43%)	10 (34.48%)	11 (39.28%)	0.032
Left superior vena cava		5 (6.25%)	1 (4.34%)	2 (6.8%)	2 (7.1%)	0.119
Patent ductus arteriosus		6 (7.5%)	2 (8.6%)	2 (6.8%)	2 (7.1%)	0.456
Double outlet right ventricle		3 (3.75%)	1 (4.34%)	1 (3.57%)	1 (3.45%)	1.457

§ =one way Anova,

# =for chi-square test

**Table 2:** Major postoperative complications.

Characteristic	Number of patients	No patch	Transannular patch	RVOT patch	*P*-value
	80 (100%)	23 (28.75%)	29 (36.25%)	28 (35.0%)	
**Postoperative complications**					
Low cardiac output syndrome	9 (11.25%)	2 (8.6%)	3 (10.34%)	4 (14.28%)	0.023
Heart block	4 (5.0%)	1 (4.34%)	2 (6.8%)	1 (3.57%)	0.058
Pleural effusion requiring tapping	3 (3.75%)	1 (4.34%)	1 (3.57%)	1 (3.57%)	1.457
Atrial arrhythmia	4 (5%)	0	2 (50%)	2 (50%)	1.00
Reoperation for bleeding	3 (3.8%)	0	2 (6.8%)	1 (3.45%)	0.001
Pulmonary regurgitation (moderate to severe)	20 (25%)	0	0	20 (25%)	0.001

## DISCUSSION

Our study looked into the results of ToF repair in adults only in terms of in-hospital mortality and complications. The survival of patients with ToF repair has greatly improved over the years. During a period from 1955 to 1968, the mortality for primary repair of ToF in the Hospital for Sick Children in Toronto was 35%, but from then onwards, the figure dropped to 12%^[5]^. The mortality rates of various cardiac centers decreased with more experience in the procedure. Dittrich et al.^[6]^ reported a mortality of 16% in the late 90's. The mortality has greatly improved in most recent reports, largely due to improved technique as well as advances in perioperative care. In the current literature, mortality as low as 0.8% and as high as 9% has been described^[7,8]^. We report a mortality of 8%, which is still high compared to mortality in paediatric age group. Dittrich et al.^[6]^ argue that this comparatively high mortality in adults may be because of the problems caused by the long-standing cyanosis. These include RV dysfunction in the form of fibrosis, cerebral complications like stroke and abscess formation and poor development of the PA. Our study does not look into the details of preoperative RV function or history of cerebral complications, but we believe that these factors play an important role in the mortality.

Various palliative procedures have been described in the literature. About 18.75% of our patients had undergone palliation procedures previously, which were taken down and a definitive repair performed. Published reports for repairs for ToF previously in Pakistan show a small number of patients being palliated alluding to the fact that the congenital cardiac surgery was in its initial stages in those days in this part of the world.

Most of the advanced centers in the world would perform a primary repair provided there are no contraindications, but there is still no consensus on this subject. Proponents of the primary repair argue that it avoids RV dysfunction and cyanotic spells which can delay developmental milestones. These patients are also prone to cerebral complications like stroke and intracerebral abscess formation. Only 15 patients in our study had undergone a previous palliative procedure and the rest underwent a primary repair in adulthood. Navabi-Shirazi et al.^[9]^ compared results of primary repair and those who had undergone some kind of palliation previously. They concluded that older patients generally do better on 2-stage repair, because of their age, but there is no conclusive data available that demonstrate that twostage repair with improvement of oxygen saturation before correction may improve outcome in this selected group of adult patients. Our study shows no mortality in the group, which had undergone palliation procedures previously.

TAP is needed for a very small-sized pulmonary valve. It carries a higher risk of reoperation, but has no impact on late survival^[10]^. We applied TAP in a comparatively high number of patients. The reason was we did not accept high degree of right ventricle/left ventricle pressure ratio and the goal was to relieve RVOT as much as possible^[11]^. Some authors have reported that the use of TAP is lower in grown patients^[12]^. Apart from a definitive indication based on size, which patient would benefit from a TAP is not fully know. Although this question would be best answered with a prospective randomized study, our experience suggests that the severity of the RVOT, rather than age at repair, is the most important determinant of the frequency of use of TAP.

TAP relieves the RVOT but, at the same time, it can cause varying degree of PR, which is associated with poor short and long-term outcome^[13]^. PR observed postoperatively in our study was 25%, which is consistent with published reports^[14]^. A large study by Kirklin et al.^[15]^ suggested that the compensatory responses to RV overload were adequate for a 20-year period, at least with respect to mortality. Our data extend these observations and do not demonstrate any difference in early survival among patients without a patch, and those with a TAP, although other investigators have implicated TAP as a risk factor for reoperation in the long term.

ToF repair in adults carry more postoperative morbidity rate compared to early repair^[16]^. Complications like low cardiac output syndrome, pericardial effusion, atelectasis, atrial arrhythmia, reoperation for bleeding and varying degree of PR were encountered in our patients. The incidence of postoperative atrial arrhythmias was higher in our adult patients compared to paediatrics as reported in the literature^[17]^. The incidence of junctional ectopic tachycardia and persistent complete heart block reported in literature is 2% and 1%, respectively^[18]^. Lungs related complications were encountered in 3.75% of our patients. Kim et al.^[19]^ reported a 5% incidence of pleural effusion that required tapping. The surgical techniques for primary repair to preserve RV function, reduce arrhythmia, and optimize functional status that are still evolving and a definitive consensus is lacking^[20]^.

Our study is not without limitations. A retrospective design and limited follow-up are some of the drawbacks. However, on the other hand, this study gives an insight to the contemporary adult cardiac surgery being practiced in Pakistan. We hope that more centers will publish their results with long-term follow-up. Especially the fate of PR and treatment strategies in our patient population needs to be ascertained. Secondly, with improvement in expertise and perioperative management in Pakistan, more and more surgeons will opt for early primary repair of ToF.

**Table:** 

Authors’ roles & responsibilities	
IK	Final manuscript approval
ZT	Realization of operations and/or trials; final manuscript approval
SA	Realization of operations and/or trials; final manuscript approval
MI	Analysis and/or data interpretation; statistical analysis; final manuscript approval
TK	Realization of operations and/or trials; final manuscript approval
AW	Final manuscript approval

## References

[1] Wald RM, Altaha MA, Alvarez N, Caldarone CA, Cavallé-Garrido T, Dallaire F (2014). Rationale and design of the Canadian Outcomes Registry Late After
Tetralogy of Fallot Repair the CORRELATE study. Can J Cardiol.

[2] Bertranou EG, Blackstone EH, Hazelrig JB, Turner ME, Kirklin JW (1978). Life expectancy without surgery in tetralogy of
Fallot. Am J Cardiol.

[3] Anderson RH, Sarwark A, Spicer DE, Backer CL (2014). Exercises in anatomy: tetralogy of Fallot. Multimed Man Cardiothorac Surg.

[4] Villafañe J, Feinstein JA, Jenkins KJ, Vincent RN, Walsh EP, Dubin AM, Adult Congenital and Pediatric Cardiology Section, American College of Cardiology (2013). Hot topics in tetralogy of Fallot. J Am Coll Cardiol.

[5] Olley PM, Kidd BSL, Keit JD (1971). Follow-up of children treated with intracardiac repair for
Tetralogy of Fallot. The natural history and progress in treatment of congenital heart
defect.

[6] Dittrich S, Vogel M, Dähnert I, Berger F, Alexi-Meskishvili V, Lange PE (1999). Surgical repair of tetralogy of Fallot in adults
today. Clin Cardiol.

[7] Lee C, Lee CN, Kim SC, Lim C, Chang YH, Kang CH (2006). Outcome after one-stage repair of tetralogy of
Fallot. J Cardiovasc Surg (Torino).

[8] Tchoumi JC, Ambassa JC, Giamberti A, Cirri S, Frogiola A, Butera G (2011). Late surgical treatment of tetralogy of Fallot. Cardiovasc J Afr.

[9] Navabi Shirazi MA, Ghavanini AA, Sajjadi S (2001). Early postoperative results after total correction of tetralogy
of Fallot in older patients is primary repair always
justified?. Pediatr Cardiol.

[10] Ylitalo P, Nieminen H, Pitkänen OM, Jokinen E, Sairanen H (2015). Need of transannular patch in tetralogy of Fallot surgery carries
a higher risk of reoperation but has no impact on late survival results of
Fallot repair in Finland. Eur J Cardiothorac Surg.

[11] Pacifico AD, Kirklin JW, Blackstone EH (1977). Surgical management of pulmonary stenosis in tetralogy of
Fallot. J Thorac Cardiovasc Surg.

[12] Vobecky SJ, Williams WG, Trusler GA, Coles JG, Rebeyka IM, Smallhorn J (1993). Survival analysis of infants under age 18 months presenting with
tetralogy of Fallot. Ann Thorac Surg.

[13] Mavroudis CD, Frost J, Mavroudis C (2014). Pulmonary valve preservation and restoration strategies for
repair of tetralogy of Fallot. Cardiol Young.

[14] Zheng DW, Shao GF, Feng Q, Ni YM (2013). Long-term outcome of correction of tetralogy of Fallot in 56
adult patients. Chin Med J (Engl).

[15] Kirklin JK, Kirklin JW, Blackstone EH, Milano A, Pacifico AD (1989). Effect of transannular patching on outcome after repair of
tetralogy of Fallot. Ann Thorac Surg.

[16] Murphy JG, Gersh BJ, Mair DD, Fuster V, McCoon MD, llstrup DM (1993). Long-term outcome in patients undergoing surgical repair of
tetralogy of Fallot. N Engl J Med.

[17] Cullen S, Celermajer DS, Franklin RC, Hallidie-Smith KA, Deanfield JE (1994). Prognostic significance of ventricular arrhythmia after repair of
tetralogy of Fallot a 12-year prospective study. J Am Coll Cardiol.

[18] Egbe AC, Uppu SC, Mittnacht AJ, Joashi U, Ho D, Nguyen K (2014). Primary tetralogy of Fallot repair predictors of intensive care
unit morbidity. Asian Cardiovasc Thorac Ann.

[19] Kim H, Sung SC, Kim SH, Chang YH, Lee HD, Park JA (2013). Early and late outcomes of total repair of tetralogy of Fallot
risk factors for late right ventricular dilatation. Interact Cardiovasc Thorac Surg.

[20] Alexiou C, Chen Q, Galogavrou M, Gnanapragasam J, Salmon AP, Keeton BR (2002). Repair of tetralogy of Fallot in infancy with a transventricular
or a transatrial approach. Eur J Cardiothorac Surg.

